# Does Cognitive Impairment Affect Rehabilitation Outcome in Parkinson’s Disease?

**DOI:** 10.3389/fnagi.2016.00192

**Published:** 2016-08-11

**Authors:** Davide Ferrazzoli, Paola Ortelli, Roberto Maestri, Rossana Bera, Nir Giladi, Maria Felice Ghilardi, Gianni Pezzoli, Giuseppe Frazzitta

**Affiliations:** ^1^Department of Parkinson’s Disease, Movement Disorders and Brain Injury Rehabilitation, “Moriggia-Pelascini” HospitalGravedona ed Uniti, Italy; ^2^Department of Biomedical Engineering, Scientific Institute of Montescano, S. Maugeri Foundation IRCCSMontescano, Italy; ^3^Movement Disorders Unit, Neurological Institute, Tel-Aviv Medical Centre, Sieratzki Chair in Neurology, Sackler School of Medicine, Sagol School for Neuroscience, Tel-Aviv UniversityTel-Aviv, Israel; ^4^Department of Physiology, Pharmacology and Neuroscience, CUNY Medical SchoolNew York, NY, USA; ^5^Parkinson Institute, Istituti Clinici di PerfezionamentoMilano, Italy

**Keywords:** Parkinson’s disease, neurorehabilitation, cognitive impairment, dysexecutive syndrome, learning

## Abstract

**Background**: The cognitive status is generally considered as a major determinant of rehabilitation outcome in Parkinson’s disease (PD). No studies about the effect of cognitive impairment on motor rehabilitation outcomes in PD have been performed before.

**Objective**: This study is aimed to evaluate the impact of cognitive decline on rehabilitation outcomes in patients with PD.

**Methods**: We retrospectively identified 485 patients with PD hospitalized for a 4-week Multidisciplinary Intensive Rehabilitation Treatment (MIRT) between January 2014 and September 2015. According to Mini Mental State Examination (MMSE), patients were divided into: group 1—normal cognition (score 27–30), group 2—mild cognitive impairment (score 21–26), group 3—moderate or severe cognitive impairment (score ≤ 20). According to Frontal Assessment Battery (FAB), subjects were divided into patients with normal (score ≥13.8) and pathological (score <13.8) executive functions. The outcome measures were: Unified Parkinson’s Disease Rating Scale (UPDRS), Parkinson’s Disease Disability Scale (PDDS), Six Minutes Walking Test (6MWT), Timed Up and Go Test (TUG) and Berg Balance Scale (BBS).

**Results**: All scales had worse values with the increase of cognitive impairment and passing from normal to pathological executive functions. After rehabilitation, all the outcome measures improved in all groups (*p* < 0.0001). Between groups, the percentage of improvement was significantly different for total UPDRS (*p* = 0.0009, best improvement in normal MMSE group; *p* = 0.019, best improvement in normal FAB group), and BBS (*p* < 0.0001, all pairwise comparisons significant, best improvement in patients with worse MMSE score; *p* < 0.0001, best improvement in patients with pathological FAB). TUG (*p* = 0.006) and BBS (*p* < 0.0001) improved in patients with pathological FAB score, more than in those with normal FAB score.

**Conclusions**: Patients gain benefit in the rehabilitative outcomes, regardless of cognition. Our data suggest that rehabilitation could be effective also in Parkinsonian subjects with cognitive impairment, as well as with dysexecutive syndrome.

## Introduction

Even with optimal pharmacological or surgical therapies, patients with Parkinson’s disease (PD) experience disabling symptoms (such as postural instability or freezing of gait) that do not respond to these treatments. Exercise is considered a possible, complementary treatment for PD (Clark et al., 1956) and recent studies showed benefits of different rehabilitative approaches on the progression of motor decay (Goodwin et al., 2008; Keus et al., 2009; Frazzitta et al., 2012, 2015b). Due to the loss of dopamine in the dorsolateral striatum, the basal ganglia region associated with the control of habitual motor behaviors, PD patients present an impaired ability to acquire and express automatic actions (Redgrave et al., 2010). Nevertheless, even if implicit learning mechanisms are defective in Parkinsonian patients in comparison to normal subjects, motor learning is feasible in PD (Nieuwboer et al., 2007) and this is crucial for rehabilitation. In this context, literature data indicate that training the patients to use cognitive strategies allows the execution of correct movements (Morris et al., 2009) under executive/volitional control.

Many PD patients present cognitive dysfunctions and the cognitive status is considered an important determinant of rehabilitation outcomes (Abbruzzese et al., 2016). However, in elderly patients with impaired cognition the effect of rehabilitation has been addressed before and several authors found that poor cognitive status does not hamper the outcomes of rehabilitative efforts (Diamond et al., 1996; Colombo et al., 2004; Heyn et al., 2004; Poynter et al., 2013). Poynter et al. (2013) in an observational study of 241 patients demonstrated that even patients with moderate cognitive impairment were able to make significant gains in grooming, dressing, toileting, transferring and mobility (as measured with Barthel Index Score) after rehabilitation treatment. Similarly, a study performed in a geriatric rehabilitation unit, on a large population, found no correlation between Mini Mental State Examination (MMSE) score and functional status on admission and demonstrated functional and clinical gains in Barthel Index Score for individuals through the full range of MMSE score (Colombo et al., 2004).

Even if this issue is very important in order to optimize the rehabilitative care, there are no data concerning the relation between cognition and rehabilitative outcome in PD.

Moving from the clinical practice with inpatient subjects with PD, in this retrospective study, we evaluated whether the cognitive decline affects the outcomes of an intensive, aerobic and goal-based rehabilitation program, based on motor-cognitive training and repetition.

## Materials and Methods

### Participants

PD patients were retrospectively identified from our Department of Parkinson’s disease, Movement Disorders and Brain Injury Rehabilitation (“Moriggia-Pelascini” Hospital—Gravedona ed Uniti, Como, Italy). Between June 2014 and September 2015 we hospitalized 752 patients suffering from different movement disorders for a 4-week Multidisciplinary Intensive Rehabilitation Treatment (MIRT; Frazzitta et al., 2012, 2013, 2015b,c). Amongst all, we excluded subjects suffering from atypical parkinsonisms (59), vascular parkinsonism (87), iatrogenic parkinsonism (10), previous stroke (7) and hydrocephalus (4). Fourty six subjects were excluded for their concomitant diagnosis of major depression or clinically significant psychiatric disorders. Moreover, 31 patients were excluded due to uncertain diagnosis and 23 did not complete the rehabilitative treatment for intercurrent medical conditions. Finally, we identified 485 subjects with PD. Parkinsonian patients were diagnosed according to the UK Brain Bank criteria (Hughes et al., 1992) and were evaluated at the beginning and at the end of the rehabilitation treatment by neurologists and physiotherapists with expertise in movement disorders field. The study design and protocol were approved by the local Scientific Committee (“Moriggia-Pelascini” Hospital, Gravedona ed Uniti—Como, Italy) and were in accordance with the code of Ethics of the World Medical Association (Declaration of Helsinki, 1967). All patients included in the study had previously given consent to use their clinical data for scientific purposes. For those patients with MMSE score <24 the informed consent had been obtained in the presence of relatives.

### Cognitive Evaluation

At the admission, a Neuropsychologist assessed the cognitive profile using the MMSE and the Frontal Assessment Battery (FAB). The tests were performed in the morning, during the medication “on” state. MMSE is a tool for screening of cognitive impairment (Folstein et al., 1975). According to MMSE normative data (Measso et al., 1993) patients were divided into three groups: Group 1 (MMSE 27–30, normal cognition), Group 2 (MMSE 21–26, mild cognitive impairment), Group 3 (MMSE ≤20, moderate or severe cognitive impairment). These cut-off scores were defined as per the UK National Institute for Clinical Excellence guidelines for classifying cognitive impairment (National Collaborating Centre for Mental Health (UK), 2007). The FAB evaluates frontal lobe dysfunctions (Dubois et al., 2000). According to FAB normative data (Appollonio et al., 2005) patients were divided into two groups: normal executive functions (FAB ≥13.8) and pathological executive functions (FAB <13.8).

### Rehabilitation Treatment

MIRT (Frazzitta et al., 2012, 2013, 2015b,c) is an intensive, aerobic and goal-based rehabilitation treatment, designed in accordance with the literature indications. All the activities included in the protocol are performed with a heart rate reserve comprised between 70 and 80%. It consists of a 4-week physical therapy, in a hospital setting, which entails four daily sessions for 5 days and 1 h of physical exercise on the sixth day. The duration of each session, including recovery periods, is about 1 h. The first session consists of a one-to-one session with physical therapist: it comprises cardiovascular warm-up activities, relaxation, muscle-stretching exercises to improve the range of motion of spinal, pelvic and scapular joints, exercises to improve the functionality of the abdominal muscles, postural changes and exercises specifically addressed to improve balance and postural control. The second session exploits the use of different devices to improve gait and balance: a stabilometric platform with visual cues (patients had to follow a pathway on a screen by using a cursor sensitive to their feet movements on the platform), treadmill plus (treadmill training with visual cues and auditory feedbacks; Frazzitta et al., 2009), crossover (Frazzitta et al., 2015a) and cycloergometer. The maximum speed of treadmill scrolling is 3.5 Km/h. The selection of the devices to adopt and the training parameters setting are defined for each patient in relation to the disease severity. The third is a session of occupational therapy to improve autonomy in everyday activities. The last session includes 1 h of speech therapy. On the sixth day patients are trained for 1 h using the devices. The rehabilitation program is personally tailored and could include: hydrokinesitherapy (for patients with severe balance and postural problems), robotic-assisted walking training for complex gait disorders, virtual-reality training and psychoeducational groups with neuropsychologists.

### Outcome Measures

Clinical, functional and motor scores were assessed at baseline and after 4 weeks by neurologists and physiotherapists with expertise in movement disorders field. All evaluations were performed in the morning, 1 h after taking drugs. The assessment included: Unified Parkinson’s Disease Rating Scale (UPDRS), Parkinson’s Disease Disability Scale (PDDS), Six Minutes Walking Test (6MWT), Timed Up and Go Test (TUG) and Berg Balance Scale (BBS).

### Statistical Analysis

The Shapiro–Wilk statistic was used to test the normality of the distribution of all variables. Most outcome variables were non-normally distributed, with severe violations to the normality assumption for BBS and TUG. Hence, to test whether the effectiveness of rehabilitation was dependent on the level of cognitive state (normal or impaired) at baseline, we considered the difference in outcome variables between the discharge and admission values and run a one-factor non-parametric analysis of variance (ANOVA) (Kruskal-Wallis) on the cognition factor (three groups for MMSE and two groups for FAB). The same analysis was carried out both on absolute and percentage differences, since both measurements provide useful and complementary clinical information. Between- and within-group comparisons were performed by the Kruskal-Wallis test and Wilcoxon signed-rank test, respectively. Following a significant result for Kruskal-Wallis test, pairwise differences in groups were analyzed by Dunn’s test (Dunn, 1964). Comparisons of categorical variables were carried out with the Chi-square test.

The association between variables was assessed by Spearman’s rank correlation coefficient.

Since a certain degree of association between cognitive state and age, years of education and Hoehn and Yahr (H&Y) was expected, multivariable regression methods were used to investigate whether changes in outcome variables were associated with cognitive state after adjustment for these covariates. Descriptive statistics for continuous variables are reported as median (lower quartile, upper quartile). Descriptive statistics for categorical variables are reported as percentage frequency. All statistical tests were two-tailed and statistical significance was set at *p* < 0.05. All analyses were carried out using the SAS/STAT statistical package, release 9.2 (SAS Institute Inc., Cary, NC, USA).

## Results

Out of 485 PD patients, according to MMSE, 236 had normal cognition (mean ± SD: 28.7 ± 1.1), 208 had mild cognitive impairment (24.8 ± 1.6) and 41 were moderately or severely cognitively impaired (17.7 ± 3.1). Considering FAB, 255 patients had normal executive functions (16.0 ± 1.4) and 230 had pathological executive functions (10.4 ± 2.6). Correlation between MMSE and FAB values was good (*r* = 0.63, *p* < 0.0001). Considering categorized values, only one patient out of the 41 with MMSE ≤20 (moderately or severely cognitively impaired) had normal executive functions (FAB ≥13.8). MMSE scores were negatively related to age (*R* = −0.38, *p* < 0.0001) and H&Y (*R* = −0.24, *p* < 0.0001) and positively related to years of education (*R* = 0.15, *p* = 0.0007). Similarly, FAB scores were negatively related to age (*R* = −0.35, *p* < 0.0001) and H&Y (*R* = −0.26 *p* < 0.0001) and positively related to years of education (*R* = 0.14, *p* = 0.002).

### Patients Grouped by MMSE

Table 1 reports demographical and clinical data of patients subdivided according to MMSE.

**Table 1:** **Demographical and clinical data of patients subdivided according to MMSE**.

	MMSE 27–30 (GROUP 1)	MMSE 21–26 (GROUP 2)	MMSE ≤ 20 (GROUP 3)
Age	65.0 (59.0, 73.0)	71.0 (67.0, 75.0)	75.0 (70.0, 78.3)
Education (years)	11.0 (8.0, 13.0)	10.0 (5.0, 13.0)	8.0 (5.0, 13.0)
H&Y scale	2.5 (2.0, 3.0)	3.0 (2.5, 3.0)	3.0 (3.0, 4.0)
L-dopa equivalent dose	617.5 (395.0, 850.0)	656.0 (430.0, 872.5)	695.0 (500.0, 830.0)
Sex (% Male)	52	61	56
Most affected side (% right)	55	50	63

*Abbreviations: MMSE, Mini Mental State Examination; H&Y, Hoehn and Yahr; L-dopa, Levodopa*.

Passing from MMSE 27–30 to MMSE 21–26 and MMSE ≤ 20, age and H&Y significantly increased as the MMSE was lower (*p* < 0.0001 both) and years of education decreased (*p* = 0.0031), while no difference was observed in levodopa equivalent dose (*p* = 0.448). No relationships between baseline score on the MMSE and sex or the most affected side were found (*p* = 0.15 and *p* = 0.28 respectively).

**Figure 1:**
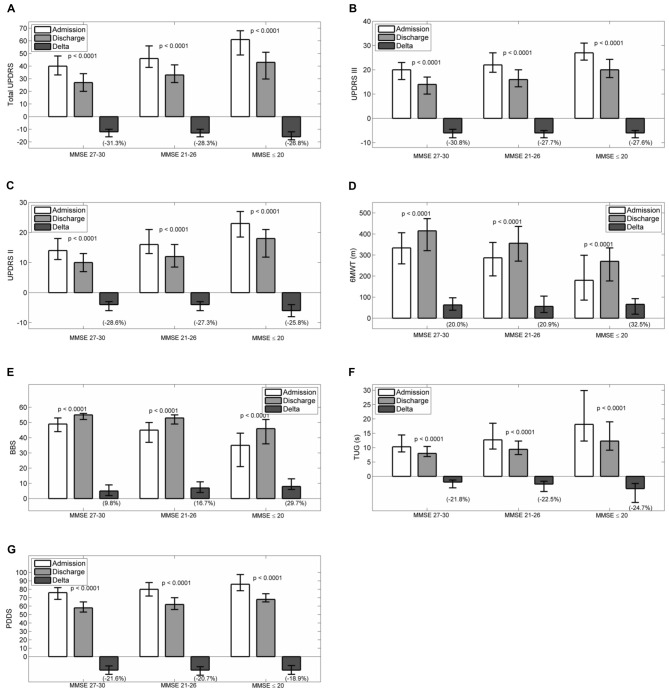
**Admission, discharge and delta (discharge-admission) for all the outcome variables in patients grouped according to MMSE: (A) Total UPDRS, (B) UPDRS III, (C) UPDRS II, (D) 6MWT, (E) BBS, (F) TUG, (G) PDDS.** Data are reported as median (box), lower and upper quartile (whiskers). The percentage value of delta is reported in brackets. The *p* values are pertaining to the comparison discharge vs. admission. Abbreviations: *MMSE, Mini Mental State Examination; UPDRS, Unified Parkinson’s Disease Rating Scale; 6MWT, Six Minutes Walking Test; BBS, Berg Balance Scale; TUG, Timed Up and Go Test; PDDS, Parkinson’s Disease Disability Scale*.

Figures 1A–G reports admission and discharge values for all outcome variables, with differences (discharge-admission) and percentage change, for every MMSE group.

At admission, all scales had worse values passing from MMSE 27–30 to MMSE 21–26 and MMSE ≤20 (total UPDRS, UPDRS II, UPDRS III, PDDS, 6MWT, BBS and TUG: *p* < 0.0001). Pairwise comparisons by Dunn’s test showed that all paired differences were significant. After rehabilitation, all scales improved significantly in each group of patients (*p* < 0.0001 all variables in all groups). To assess whether the degree of improvement was different between groups, the percentage improvements in outcome variables were compared. Significant differences were observed for the changes in total UPDRS (*p* = 0.0009, no pairwise significant differences and best improvements in patients with normal MMSE score), PDDS (*p* = 0.025, with significant differences between MMSE ≤20 and MMSE 27–30 and best improvements in patients with normal MMSE score), UPDRS III (*p* = 0.0041, with significant differences between MMSE ≤20 and MMSE 27–30 and MMSE 21–26 and MMSE 27–30 and best improvement in group 1 or 2), BBS (*p* < 0.0001, all pairwise comparisons significant and best improvement in patients with moderate or severe cognitive impairment).

For the remaining outcome variables, namely UPDRS II, 6MWT and TUG no between groups significant differences were observed in the percentage changes (*p* = 0.211, *p* = 0.159, *p* = 0.277, respectively).

### Patients Grouped by FAB

The same analysis was carried out dividing patients according to FAB. Table 2 reports basal values for demographical and clinical variables in patients with normal executive functions (FAB ≥13.8) and pathological executive functions (FAB <13.8).

**Table 2:** **Demographical and clinical data of patients subdivided according to FAB**.

	FAB ≥ 13.8	FAB < 13.8
Age	66.0 (59.0, 72.0)	71.0 (67.0, 77.0)
Education (years)	12.0 (8.0, 13.0)	8.0 (5.0, 13.0)
H&Y scale	2.5 (2.0, 3.0)	3.0 (2.5, 3.0)
L-Dopa equivalent dose	600.0 (392.5, 850.0)	665.0 (458.8, 876.3)
Sex (% Male)	54	58
Most affected side (% right)	53	54

*Abbreviations: FAB, Frontal Assessment Battery; H&Y, Hoehn and Yahr; L-Dopa, Levodopa*.

Passing from normal to pathological executive functions, age and H&Y significantly increased (*p* < 0.0001 both), years of education decreased (*p* = 0.0009), while no difference was observed in levodopa equivalent dose (*p* = 0.073). No different distribution of sex and most affected side was found (*p* = 0.290 and *p* = 0.840 respectively).

**Figure 2:**
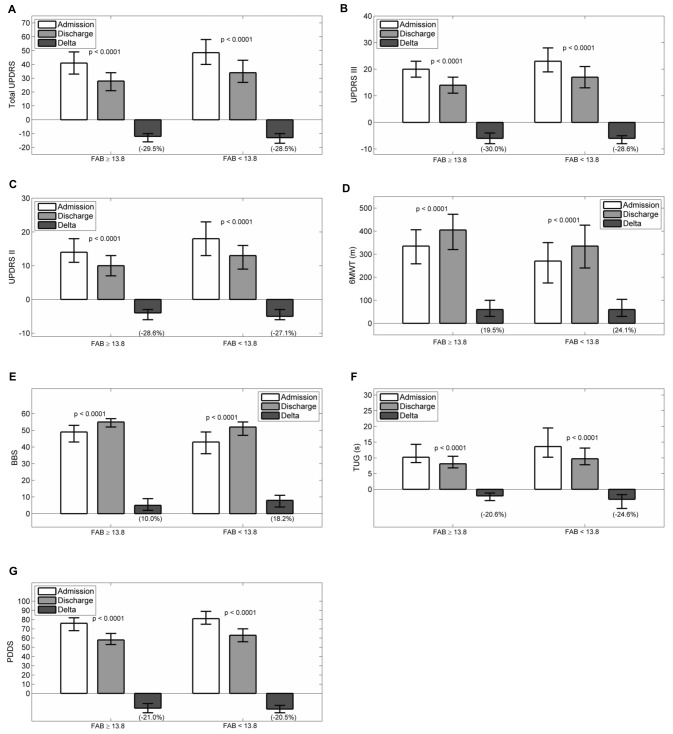
**Admission, discharge and delta (discharge-admission) for all the outcome variables in patients grouped according to frontal assessment battery (FAB): (A) Total UPDRS, (B) UPDRS III, (C) UPDRS II, (D) 6MWT, (E) BBS, (F) TUG, (G) PDDS.** Data are reported as median (box), lower and upper quartile (whiskers). The percentage value of delta is reported in brackets. The *p* values are pertaining to the comparison discharge vs. admission. Abbreviations: *MMSE, Mini Mental State Examination; UPDRS, Unified Parkinson’s Disease Rating Scale; 6MWT, Six Minutes Walking Test; BBS, Berg Balance Scale; TUG, Timed Up and Go Test; PDDS, Parkinson’s Disease Disability Scale*.

Figures 2A–G reports admission and discharge values for all outcome variables, with differences and percentage change, for both groups.

At admission, all scales had worse values passing from normal to pathological executive functions (total UPDRS, UPDRS II, UPDRS III, PDDS, 6MWT, BBS and TUG: *p* < 0.0001). After rehabilitation, all scales improved significantly in both groups (*p* < 0.0001 all variables in both groups). Comparing the percentage improvements in outcome variables in the two groups, significant differences were observed only for changes in total UPDRS (*p* = 0.019, best improvement in patients with normal FAB score), BBS and TUG (*p* < 0.0001 and *p* = 0.006 respectively, best improvement in patients with pathological FAB score).

Finally, no significant difference in the percentage changes was observed for UPDRS III, UPDRS II, 6MWT and PDDS, (*p* = 0.111, *p* = 0.103, *p* = 0.06 and *p* = 0.432 respectively), indicating a similar beneficial effect of rehabilitation in the two groups as assessed by these outcome variables.

### Role of Covariates

Since age, H&Y and education were strongly related to cognition status, we also investigated the relationship between improvement in outcome variables and cognition status after correction for these covariates. Considering patients grouped according to MMSE, only percentage changes in total UPDRS and BBS remained significantly different between the three groups after correction (*p* = 0.04 and *p* = 0.008 respectively), while considering patients grouped according to FAB no percentage changes remained significantly different between the two groups after correction. However, these significant differences don’t appear to have clinical relevance.

## Discussion

This is the first study that addresses the issue of the relationships between cognitive impairment and rehabilitative outcome in PD patients. As opposed to what is normally believed (Abbruzzese et al., 2016), our data demonstrates that also individuals with cognitive impairment treated with a specific, intensive rehabilitative approach were able to gain comparable benefits in motor and functional outcomes as patients without cognitive impairment. Since there are several PD patients with cognitive deficits, it is important to re-examine the question of rehabilitative care also for these subjects. In our patients, age, severity of disease (evaluated with H&Y scale) and cognition (evaluated with MMSE and FAB) were correlated with each other: higher age and more advanced motor disturbances were associated with poorer cognitive performances. In accordance with literature data (Kuzis et al., 1999; Appollonio et al., 2005), MMSE and FAB scores showed a direct relation with years of education and an inverse relation with age. In all groups, no differences were found in the total amount of levodopa dose. This finding allows us to exclude a secondary, dose-dependent effect of dopaminergic therapy on rehabilitative outcomes. According to the patients’ subdivision on the basis of MMSE score, we observed that the three groups differ at the baseline in all scales. These differences have a relation with cognition: with the worsening of MMSE score, the performances get worse. This finding could be explained with the worsening of performances as age increases or with the existence of a direct relation between cognition and motor skills. However, even after correction for age, disease severity and years of education, cognitively impaired patients obtain significant benefits after rehabilitation.

Since PD patients frequently suffer from dysexecutive syndrome, we evaluated also the impact of this condition on rehabilitation subdividing patients on the basis of FAB score. Even in this case, in the group with pathological score the performances at baseline were worse and both groups improved in all scales after rehabilitation. Moreover, data concerning the improvement obtained in TUG and BBS score by patients with dysexecutive syndrome has a very important clinical relevance: these patients had pathological scores at admission and get normal performances after the treatment (Qutubuddin et al., 2005; Nocera et al., 2013).

We found that rehabilitation is effective in PD patients with cognitive impairment, as much as in those with normal cognition. Indeed, even if the percentage improvement in total UPDRS was significantly greater in patients with preserved cognition, all groups showed a significant improvement after rehabilitation.

Our findings could indicate that even in presence of cognitive decline, the ability to learn is not completely hindered. We argue that our intensive and goal-based rehabilitation treatment, entailing constant and repetitive exercises, exploits both the implicit and the explicit learning. Implicit learning is the acquisition of knowledge without awareness and volition (Reber, 1989) and differs from explicit learning, defined as a form of conscious and intentional process of knowledge acquisition (Cohen et al., 1985). Striatum plays a central role in implicit learning (Gamble et al., 2014), the anterior cingulate/mesial prefrontal cortex supports the explicit component of learning, whereas hippocampus is crucial for both explicit and implicit processes (Gamble et al., 2014). People with PD have degeneration of dopamine-producing neurons, leading to dopamine deficiency, striatal deficits and consequent impairment in implicit learning (Rieckmann et al., 2010; Gamble et al., 2014). Moreover, several studies demonstrated that patients suffering from PD with dementia have deficits on tasks of explicit learning as well as on some task of implicit learning (Bondi and Kaszniak, 1991; Kuzis et al., 1999).

It is conceivable that the proposed treatment is able to optimize motor learning both explicit, exploiting the acquisition of voluntary strategies, and implicit through incremental stimulus-response associations.

Therefore, MIRT probably works through the interaction between implicit and explicit learning mechanisms. This interaction was theorized and described previously: Destrebecqz et al. (2005) found that anterior cingulate/mesial prefrontal cortex exerts control on the activity of the striatum and suggested the existence of a functional interaction between different brain regions that subtend different computational objectives and in which information processing appears to be either accompanied by awareness (the anterior cingulate/mesial prefrontal cortex) or not (the striatum).

Probably, other mechanisms underlie the improvement we found after the treatment. In particular, with respect to walking, spinal mechanisms that are not influenced by cognition could be implicated. It has been postulated that treadmill training could provide adequate sensory inputs, which may stimulate the spinal locomotor circuitry (Van de Crommert et al., 1998). Certainly, treadmill-plus through the use of feedbacks and cues optimize learning. However, the belt of the treadmill itself forces the subject to step, through stretch facilitation of hip flexors and ankle plantar flexors and provides adequate sensory inputs which stimulates the spinal locomotor circuitry, stressing the functioning of the Central Pattern Generator (Bello and Fernandez-Del-Olmo, 2012). Beyond these mechanical concepts, Edgerton et al. (1991) provided evidence that different strategies, including treadmill training, could produce functional changes or “motor learning” properly in the spinal motor-generating circuitry.

Finally, we found better percentage of improvement in TUG and BBS in patients with pathological FAB score. Dysexecutive syndrome negatively affects those motor performances that entail a high degree of executive components, like TUG and BBS. Since any task that involves planning and executing a goal-directed action requires executive functions (Morris et al., 2001), we explain these results hypothesizing that MIRT leads to an improvement in these scales through the voluntary/attentive application of learned motor skills.

There are some limitations to this study: first, we did not perform a detailed assessment of neuropsychological functions. This would allow us to understand better the cognitive profile of patients and evaluate this in relation to the rehabilitative outcomes. We did not consider the aspect of the generalization of learned abilities. Further studies should explore how patients with and without cognitive decline transfer the effect of the treatment in daily life and the duration of the obtained benefits over time. Moreover, different rehabilitation approaches have been proposed for patients with PD: in this study we evaluated only the efficacy of an intensive and goal-based treatment. This is not enough to affirm that is possible to achieve the same results with other, different rehabilitation approaches. Further studies are needed to clarify this issue.

## Conclusion

We demonstrated for the first time that PD patients in early, medium and advanced stages of disease with cognitive dysfunctions, who undergo a specific treatment exploiting implicit learning strategies, are able to gain benefit in rehabilitative outcomes. Our data allow challenging the belief that rehabilitation should be reserved only for patients with normal cognition.

## Author Contributions

DF wrote the text, provided substantial contributions to discussion of the content and edited the manuscript before submission; PO wrote the text, provided substantial contributions to discussion of the content and researched data for the article; RB researched data for the article; RM did the statistical analysis and generated tables and figures; NG, GP, MFG did a critical revision and provided substantial contributions to discussion of the content; GF wrote the text, provided substantial contributions to discussion of the content and did a critical revision.

## Conflict of Interest Statement

DF, PO, RM, RB, MFG, GP, GF declare no disclosures or conflict of interests. NG—Prof. Giladi serves as a member of the Editorial Board for the Journal of Parkinson’s Disease. He serves as consultant to Teva-Lundbeck, Intec Pharma, NeuroDerm, Armon Neuromedical Ltd./Dexel, Monfort and Lysosomal Therapeutic Inc. He received payment for lectures at Teva-Lundbeck, Novartis, UCB, Abviee, Shaier and Genzyme. Prof. Giladi received research support from the Michael J Fox Foundation, the National Parkinson Foundation, the European Union 7th Framework Program and the Israel Science Foundation as well as from Teva NNE program, LTI, and Abviee and CHDI. The funders had no role in study design, data collection and analysis, decision to publish, or preparation of the manuscript. There are no patents, products in development or marketed products to declare. This does not alter our adherence to all the *Frontiers in Aging Neuroscience* policies on sharing data and materials, as detailed online in the guide for authors.
